# Habitat and Season Effects on Small Mammal Bycatch in Live Trapping

**DOI:** 10.3390/biology11121806

**Published:** 2022-12-13

**Authors:** Ines Hotopp, Bernd Walther, Olaf Fuelling, Daniela Reil, Christin Hesse, Diana Alexandra Below, Christian Imholt, Jens Jacob

**Affiliations:** 1 tier3 Solutions GmbH, Kolberger Str. 61–63, 51381 Leverkusen, Germany; 2 Rodent Research, Institute for Epidemiology and Pathogen Diagnostics, Julius Kühn-Institute (JKI)–Federal Research Centre for Cultivated Plants, Toppheideweg 88, 48161 Münster, Germany

**Keywords:** conservation, non-target species, endangered species, rodents, Ugglan traps, voles

## Abstract

**Simple Summary:**

Trapping particular small mammal species is frequently used for scientific purposes but unnecessary bycatch can occur. Live trapping conducted over the last decade in Germany using Ugglan multiple capture traps in grassland, forest and margin habitats revealed about 30% bycatch when target species were common voles (*Microtus arvalis*) in grassland and common voles and bank voles (*Clethrionomys glareolus*) in margins and forests. This was more pronounced in spring and along margins. Species mentioned on the early warning list according to the Red List Germany were higher in numbers and proportion in spring and in grassland. The results of the study will help to avoid periods with enhanced presence of bycatch including endangered species (if the purpose of the study allows) or to pay particular attention in certain seasons and habitats when the occurrence of bycatch is most likely.

**Abstract:**

Trapping small mammals is frequently used to study the dynamics, demography, behavior and presence of pathogens. When only particular small mammal species are in the focus of interest, all other species are unnecessary bycatch. We analyzed data from extensive live trapping campaigns conducted over the last decade in Germany, following a consistent standard trapping protocol that resulted in about 18,500 captures of small mammals. Animals were trapped with Ugglan multiple capture traps in grassland, forest and margin habitat. Trap success and the proportion of bycatch were about 30% when target species were common voles (*Microtus arvalis*) in grassland and common voles and bank voles (*Clethrionomys glareolus*) in margins and forests. This was more pronounced in spring and along margins. Species mentioned in the early warning list according to the Red List Germany were higher in numbers and proportion in spring and in grassland. The results will help to avoid periods with enhanced presence of bycatch, including endangered species (if the purpose of the study allows) or to pay particular attention in certain seasons and habitats when the occurrence of bycatch is most likely.

## 1. Introduction

Capturing wildlife is important to humans as wild animals are recognised as a source of nutrients and material for shelter, tools, medicine and cultural objects, etc. For millennia, wild animals have been vital in the process of domestication, for support in hunting and herding, lifting and transporting objects and other work, religious rites and as guards, as well as companions. The importance of wildlife in these aspects was later supplemented by the relevance of wildlife in research. In many cases, the species required needs to be obtained.

While capture for food does not necessarily require the animal to stay alive, their use for observational studies does. In some aspects of research such as the study of animal populations, there is an obvious need not to disturb the object of study by unduly removing individuals [1]. In the last few decades, more and more emphasis has been put on the welfare of wildlife in research, including when trapping animals in the wild [2,3]. For research purposes, target animals should be placed in optimal conditions during the process of capturing, handling and releasing or transferring to research facilities, not only to ensure the welfare of animals [4] but also to ensure the validity of the research results obtained with them [5].

Rodents are the most species-rich mammalian order, with more than 2500 recent species [6]. Today, rodents are used for human [7,8] and animal food [9], pelts [10], entertainment [11], as pets [12,13] and, probably most importantly, for research [14,15]. Some rodent species are significant pests [16] and some can host and transmit zoonotic diseases to humans, pets and livestock [17]. Several rodent species are (critically) endangered or vulnerable [18].

Rodents are by far the vertebrate taxon most often bred and captured for research purposes. Wild rodents are obtained by live trapping and transferred to holding facilities (laboratories, enclosures) for various studies of their biology and ecology, including behavioural aspects [19,20] and risk assessment [21,22]. Others are trapped and released in situ for monitoring population dynamics [23,24], dispersal [25] or the epidemiology of rodent-borne pathogens [26,27].

Small mammals of a similar size to target species such as passerine birds, amphibians and reptiles are reported as bycatch in kill trapping [28,29] and live trapping [30,31]. Such bycatch can yield additional information as a rare, invasive and/or unseen/new species may be detected, but studies considering particular target species usually aim to minimize bycatch. While the death of animals is inevitable by design in kill traps, target and non-target animals may also die in live traps [32,33]. This can be due to fatal stress, bait that cannot be utilised by bycatch species or aggressive encounters between target and non-target animals caught in the same trap [34,35].

Attempts made to minimise bycatch include the choice of bait [36,37] and trap type most suitable for the target species [38,39], as well as optimal trapping location [40,41], optimal spacing [42,43] and signalling devices that allow for swift trap checks after capture [44]. In addition, modifications are made to reduce the accessibility of traps for non-target species such as a cover to exclude birds [45], trigger weight adjusted to the weight of the target species [46] or structures for escape [30] such as a “shrew hole” to let shrews escape [47,48].

Alternative methods to trapping that prevent bycatch include structures to sample hair [49,50] or scats [51], wildlife cameras [52] or indices of abundance that do not rely on trapping such as burrow counts [53], track counts [54,55], genetic methods based on non-invasive sampling methods [56] or chew cards and monitoring blocks [55,57].

However, in many circumstances, live trapping is the gold standard and often cannot be replaced by alternative methods. With live traps, often more individuals and more species can be captured than with snap traps [58,59]. Therefore, live trapping will remain the main method in studies of small mammals, be it for monitoring or the investigation of their population dynamics, demography or behaviour, while the study of associated pathogens often requires organ samples and the use of kill traps.

There is only limited knowledge through systematic and comprehensive long-term study of bycatch in live trapping. We analysed bycatch in live trapping data collected in Germany using Ugglan multiple capture traps over the last decade. Target species were the forest-dwelling bank vole (*Clethrionomys glareolus* syn. *Myodes glareolus*) and the grassland species common vole (*Microtus arvalis*). Both are small rodent species that are widely distributed across large parts of Europe [60] and regions in the western part of Asia [6]. The multi-annual dynamics are similar and characterised by population outbreaks every 2–5 years [61]. During outbreaks, common voles cause massive damage in agriculture and forestry throughout Europe [62], while large bank vole populations can cause damage in forestry [61] and increase the risk of transmission of Puumala orthohantavirus to humans [63,64]. For a better understanding of the patterns and processes related to outbreaks, and to inform stakeholders in plant protection and health protection about the risks associated with outbreaks of these species, research and monitoring using live trapping are conducted regularly. Here, data from past trapping campaigns were re-analysed in order to gain a better understanding of patterns of the occurrence of bycatch. The effects of habitat, season and Red List status of non-target species were considered to identify when and where researchers should pay particular attention to minimise bycatch.

## 2. Materials and Methods

### 2.1. Field Sites

**Figure 1:**
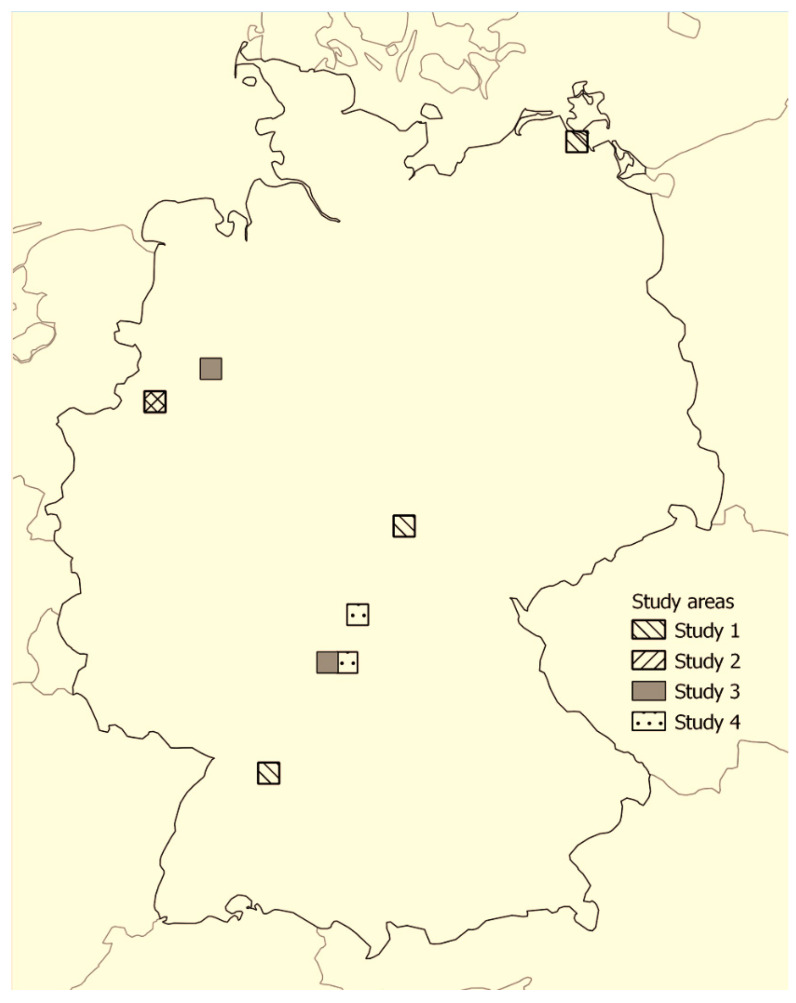
Location of study sites in Germany. © map shape: UIA World Countries Boundaries https://hub.arcgis.com/maps/252471276c9941729543be8789e06e12 accessed 2 December 2022.

In four different studies, we used study sites totalling 28 forest, 8 grassland and 23 field margin habitats in six federal states of Germany (Table 1, Figure 1). All sites were at least 0.2–2.2 km apart from each other. Forest habitats were mainly beech forests or mixed deciduous forests dominated by beech (*Fagus sylvatica*), oak (*Quercus* spp.) and hornbeam (*Carpinus betulus*). Some forest sites were located at forest edges with dense understory vegetation. Grasslands were sown with perennial commercial seed mixes mainly used to produce fodder, including silage for dairy cattle or for grazing livestock. Margins were mostly hedgerows consisting of various woody species and shrubs that separated different arable fields or grasslands.

### 2.2. Trapping

We used Ugglan live trap models No. 1 and No. 2 (Grahnab, Gnosjö, Sweden) for trapping small mammals between March and November in 2010–2015 and 2019–2021. The Ugglan multi-capture live trap is frequently used in small mammal research all over Europe (e.g., [24,39]). The trap is made from wire mesh with a removable plastic floor for insulation and easy cleaning and an aluminium cover for weather protection. The trap is large enough to hold more than one small mammal and is usually equipped with bait and bedding. In studies 1 and 2 (Table 1), we arranged Ugglan live traps in grids of 7 × 7 (49) and 5 × 10 (50) traps with trap spacing of 7–10 m. In studies 3 and 4, we used 20–30 traps in 2–4 trap-lines 2–10 m apart and trap spacing of 10 m within lines. We pre-baited traps for 1–3 days before activating traps. In studies 1–2, bait was rolled oats supplemented with peanut curls, apple slices and rodent chow pellets and bedding was wood wool. In studies 3–4, bait was rolled oats, olive oil and dried mealworm supplemented with slices of apple, cucumber or carrot when temperatures were high and bedding when temperature was low.

In studies 1 and 2, live traps were set for three to four consecutive days and checked every 10–12 h, always around sunrise and sunset. In studies 3 and 4, we placed live traps for three consecutive nights, and set them each night for about six hours around sunset or sunrise. Within the six hours, taps were set and checked every 90–120 min. Animals were released at the point of capture. For the purpose of this analysis, we defined the bank vole and the common vole as the target species in forests and margins and the common vole as the target species in grassland. Both species are important model species in various scientific studies and in monitoring to assess the risk of rodent damage in forestry and agriculture or the risk of transmission of Puumala Orthohantavirus from bank voles to humans. However, the original studies might have targeted not just bank voles or common voles but other small mammal species as well. All other captured species were defined as non-target species for the present analysis.

### 2.3. Data Analyses

We analysed 18,458 captures of small mammals from 59 study sites obtained in 83,355.5 trap nights in nine years (Table 1). Only trapping occasions that yielded ≥ 5 captures were considered for analyses. We defined trap night as a period of approximately 12 h between trap checks independent of the time of the day (studies 1 and 2). A period of six hours between trap checks as in studies 3 and 4 was regarded as 0.5 trap nights.

The number of captures and the proportion per taxon, site and session was calculated as well as the number (captures per 100 trap nights [TN] [65] rounded to integers) and the proportion of non-target species in captures for each study site and trapping session. The Red List Germany (https://www.rote-liste-zentrum.de/en/Download-Vertebrates-1874.html accessed on 20 December 2021) [66] was used to allocate taxa according to Red List status. The proportion of captures of species on the early warning list (for simplicity labelled “endangered” throughout the manuscript) per site and session was calculated.

The effect of season and habitat on trap success and proportion of captures per taxon, non-target captures and red list status were assessed with program R, Vers. 4.1.1 [67]. Effects of the ecological factors habitat (forest, grassland, margin) and season (spring, summer, autumn) were analysed using generalised linear mixed models (GLMMs). Models were fitted by maximum likelihood estimation via Template Model Builder and Laplace approximation for the integration of random effects using the R-package glmmTMB, Vers. 1.1.2 [68]. If necessary, a zero-inflation model was included. Proportions were fitted with a binomial family and trap success was fitted using a negative binomial family to account for overdispersion in the count data. For each parameter, the model with the best fit according to the Akaike’s information criterion (AIC) [69] for fixed and random effect structures and, if applicable, zero-inflation formula was selected. The validity of the chosen models was checked by graphical evaluation of the normality of the residuals, the linearity between response and predictors, the independence of residuals and the calculation of the variance inflation factors (VIFs). As the models contained interaction terms, VIFs could be expected to be high for some terms.

The final GLMMs for the proportion of non-target species included the explanatory variables habitat and season and their interaction. Year and study site nested per study were selected as random intercepts to account for repeated measurements and possible clustering of data along these factors. To assess the proportion of endangered species, the final GLMM included the interaction of all explanatory variables (habitat, season) and the random intercepts (year and study site). Figures were produced with the R-package ggplot2, Vers. 3.3.5 [70].

There were differences in the aim and design of studies 1–2 vs. 3–4 (see above). Studies 3–4 focussed on shrews in margins in spring and summer with frequent trap checks. This resulted in higher trap success, more Cricetidae and more non-target captures than in studies 1–2 that also covered seasons with low occurrence of Cricetidae. For the statistical analysis this is not ideal because even a simple calculation such as the mean per season can be skewed. An apparent temporal trend indicating low trap success in autumn is deceptive, because it is simply due to the missing data from studies 3–4. However, GLMMs are able to incorporate such misbalanced data. The study was included as a random effect and therefore the model includes the variability between the studies in calculations and predictions. Therefore, the results of the analysis of trap success per taxon and of non-target species captures are presented based on the model predictions and not as mean values. For the other variables, arithmetic means ±standard errors are reported throughout. For all analyses, the level of statistical significance was set to α < 0.05.

## 3. Results

During live trapping sessions in forest, grassland and margins, 18,458 small mammals of at least 15 species of four mammalian families were recorded (Table 1 and Table 2). No other vertebrate species was trapped.

Trap success (captures per 100 TN) was highest in margins (40.0), followed by forests (22.3) and grasslands (13.3). In spring, the overall trap success was lowest (18.0) and it was highest in summer (26.7, autumn: 21.3) (Table 2 and Table 3). In combination with habitat and season, trap success was highest in margins in spring (62.9) and lowest in grassland in spring (5.7) (Table 3).

**Figure 2:**
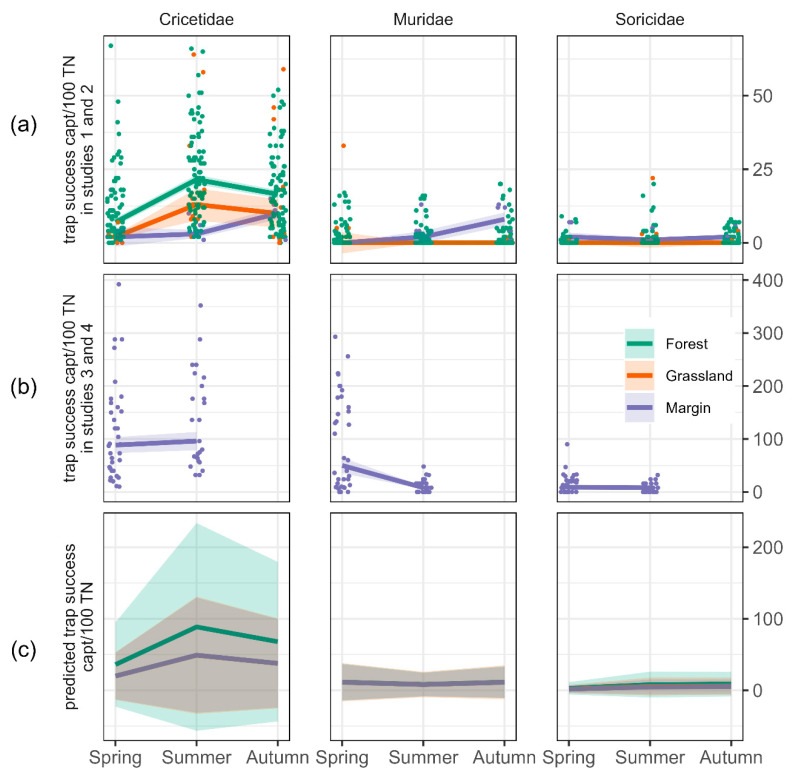
(**a**) Mean trap success (captures per 100 trap nights) per taxon, habitat and season in studies 1 and 2, (**b**) mean trap success (captures per 100 trap nights) per taxon, habitat and season in studies 3 and 4. Coloured areas in (**a**,**b**) depict the standard error. Means are based on multiple sites and trapping occasions (coloured dots). Means are presented separately for studies 1–2 and 3–4 to account for the otherwise skewed presentation due to varying study designs. (**c**) Predicted trap success by a GLMM with negative binomial family. Coloured areas in (**c**) depict the confidence interval of the statistical model. Note the different scales of the y-axes.

On average, 34.1 (±2.8) Cricetidae, 11.6 (±2.0) Muridae and 3.0 (±0.4) Soricidae were captured per 100 trap nights. The data contained many zeros and therefore the GLMM included a zero-inflation model. The model showed that Cricetidae were trapped twice as often as Muridae (*p* < 0.05) and 4.5 times as often as Soricidae (*p* < 0.05). Cricetidae were trapped 1.8 times more often in forests than in grasslands and margins (*p* < 0.05) (Figure 2a–c). For Muridae and Soricidae, trap success did not differ between habitats. According to model predictions, trap success of Cricetidae was 2.5 times higher in summer and in autumn 1.9 times higher than in spring (Figure 2a–c). In contrast, trap success of Muridae in summer was half as high as in spring (*p* < 0.05), but was similar in spring and autumn. Trap success of Soricidae in summer and autumn was at least 1.5 times higher than in spring (*p* < 0.05). Soricidae were more likely to be completely absent than the other two taxa (*p* < 0.05), and more likely to be completely absent in spring than in summer or autumn (*p* < 0.05).

**Figure 3:**
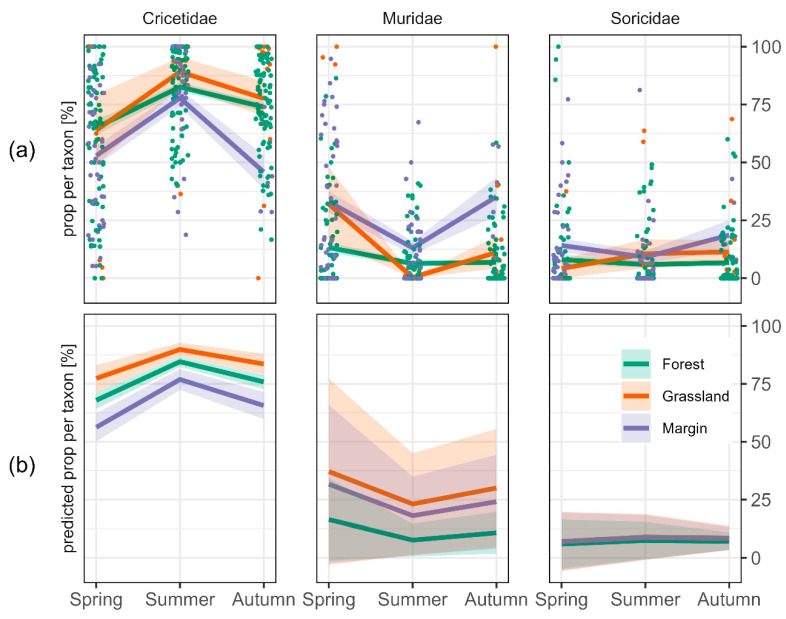
(**a**) Mean values for the proportion of captures per taxon, habitat and season. Coloured areas depict the standard error. Means are based on multiple sites and trapping occasions (coloured dots), and (**b**) predicted proportions by a GLMM with binomial family. Coloured areas in (**b**) depict the confidence interval of the statistical model.

The average proportion of Cricetidae was more than 5-fold higher than of Muridae and Soricidae (*p* < 0.05). It was highest in grassland and significantly lower in forests and margins (Figure 3a,b). A peak was reached in summer with a 1.3-fold increase (*p* < 0.05) compared to spring, but proportions were also significantly higher in autumn than in spring (1.2-fold increase) (Figure 3a,b). The proportion of Muridae was lowest in forests. In grassland, it was 1.4-fold increased (*p* < 0.05), and in margins 2.9-fold (*p* < 0.05) (Figure 3b). The proportion of Soricidae did not differ significantly among habitats, but was >15% lower in summer and autumn than in spring (Figure 3b). The likelihood of absence of Muridae and Soricidae was higher than for Cricetidae. For Soricidae it was lowest in spring (*p* < 0.05).

**Figure 4:**
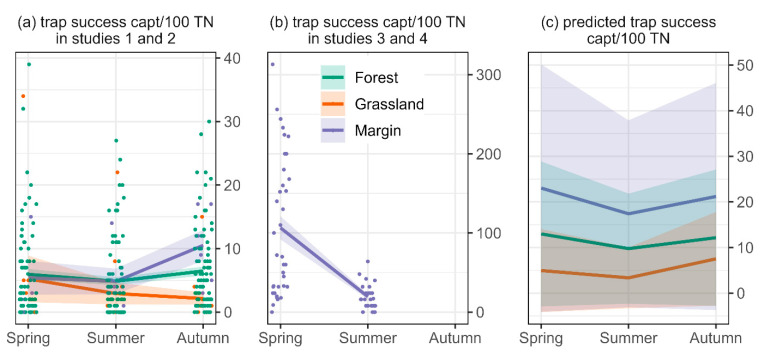
(**a**) Mean trap success of non-target captures (captures per 100 trap nights) per habitat and season in studies 1 and 2, (**b**) mean trap success of non-target captures (captures per 100 trap nights) per habitat and season in studies 3 and 4. Coloured areas in (**a**,**b**) depict the standard error. Means are based on multiple sites and trapping occasions (coloured dots). Means are presented separately for studies 1–2 and 3–4 to account for the otherwise skewed presentation due to different study designs. (**c**) Predicted trap success with a GLMM with negative binomial family. Coloured areas in (**c**) depict the confidence interval of the statistical model. Note the different scales of the y-axes.

Trap success of non-target species (16.4 captures per 100 TN ± 2.1) was on average approximately half of the trap success of target species (34.0 captures per 100 TN ± 2.9). Model predictions suggested similar trap success of non-target species for margins, forest and grassland and for seasons (Figure 4a–c). The absence of non-target species was more likely in grassland than in forests and in spring than in autumn (*p* < 0.05) (Figure 4a–c).

**Figure 5:**
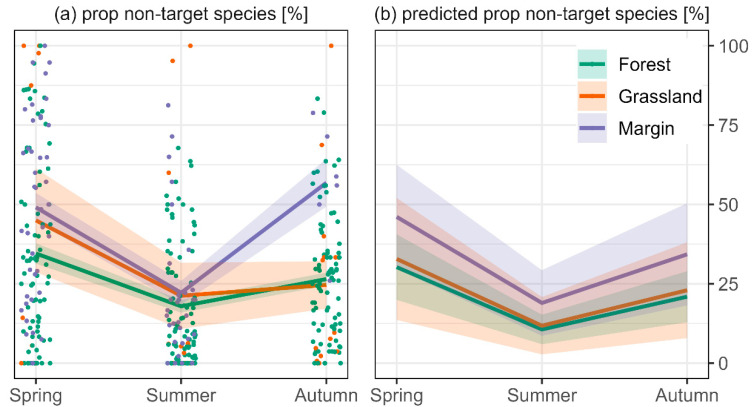
(**a**) Mean proportion of non-target captures per habitat and season. Coloured areas depict the standard error. Means are based on multiple sites and trapping occasions (coloured dots), and (**b**) predicted proportion of non-target species by GLMM with binomial family. Coloured areas in (**b**) depict the confidence interval of the statistical model.

The average proportion of non-target species captures was 0.71 (±0.01). The proportion of non-target species captures was 50% higher in margins than in forest (*p* < 0.05) but did not differ between grassland and forest (Figure 5a,b). It was 100% higher in spring than in summer (*p* < 0.05) and 30% lower in autumn than in spring (Figure 5a,b) (*p* < 0.05).

**Figure 6:**
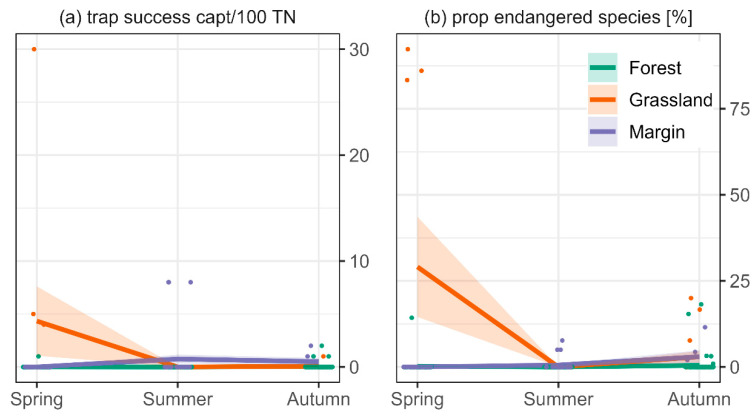
(**a**) Mean number (captures per 100 trap nights) and (**b**) mean proportion of endangered species (according to Red List Germany [66]) that were captured per habitat and season. Coloured areas in (**b**) depict the standard error. Means are based on multiple sites and trapping occasions (coloured dots). Note the different scales of the y-axes.

The occurrence of endangered species (*Micromys minutus* and *Neomys fodiens*) trapped in the studies was too scarce for a thorough statistical analysis. Available data suggest an increased presence of endangered species in grassland compared to margins (3.2-fold increase) and forests (54-fold increase) (Figure 6a) and higher trap success in spring compared to summer (1.75-fold increase) and autumn (4.3-fold increase) (Figure 6a). Similarly, the proportion of endangered species seemed highest in grassland and in spring (Figure 6b).

## 4. Discussion

To our knowledge, this is the first attempt to investigate live trapping data from central Europe regarding the distribution of captures of target and non-target taxa across habitats, Red List status and season. The study shows that, overall, about a third of live trapped small mammals were bycatch. This is not surprising because the small mammal composition of central European deciduous forests comprises a variety of small mammal species [71] dominated by bank voles and *Apodemus* species [72,73]. The same is true for open landscape habitats adjacent to hedgerows, forests and human settlements, where common voles are most prevalent [74,75].

The considerable proportion of non-target animals in standard live trapping is unwanted but less worrying than in kill trapping because animals can be released. However, there can be mortality in small mammals trapped alive [32,33]. Bycatch is also known from trapping programs of other taxa such as invertebrates [76,77] and birds [78]. Insect bycatch can be reduced using species-specific pheromones as lures [79] and specific birds such as starlings (*Sturnus vulgaris*) can be attracted by placing a conspecific individual close to the trapping device or using species-specific bird calls [80]. Species-specificity cannot be achieved with Ugglan traps considered here or with other standard live traps used in small mammal research such as Longworth traps or Sherman traps because they all, by design, capture all animals small enough to pass through the trap’s entrance [39,81].

Generally, the trap success of non-target taxa was higher in forest versus margins and grassland and higher in spring than summer or autumn. Naturally, the more restricted the number of target species, the higher the proportion of non-target species trapped. We have used an extreme approach focussing on only 1–2 target species in each habitat, albeit rather common ones. Therefore, the proportion of non-target species we report is likely to be drastically reduced when several species are targets.

Habitat mattered for bycatch because trap success of non-target species was higher in margins than in forest habitats. This finding seems plausible as in central Europe, open agricultural habitats including grassland that stretch far between margins are of poor small mammal diversity. Grassland tends to host the common vole as the main species with few other small mammals present [75,82]. The woody plants and shrubs in margins provide more complex horizontal and vertical structures that can be a suitable habitat or provide a dispersal corridor for several small mammal species [71,83]. Non-targets were obviously attracted to the live traps at habitat interfaces, leading to an elevated proportion in margins.

Seasonally, the proportion of non-target taxa was highest in spring independent of habitat. Several factors may have caused this result. Spring temperatures are lower than in summer and autumn [84], body condition after winter tends to be low [85,86] and age of animals high [87], which may all cause increased need for food uptake. However, in spring, food availability is low. This could have caused non-target species more than target species to utilise bait in traps more intensely than they might do in other seasons. Such species may also tolerate the (previous) presence of other species in and around traps in spring more than in other seasons [88,89]. Additionally, limited food supply in spring may prompt non-target species to accept potential predation risk associated with increasing home rages and approaching traps in an otherwise less preferred landscape of risk [90,91]. All this could result in increased captures of non-target species but this needs to be validated in future work.

We used data from previous studies that were not designed a priori to assess bycatch and therefore were slightly different in bait used, trapping schedule and trap type. However, the large numbers of trap nights and captures yielded a robust indication where main non-target issues can occur. It seems unrealistic to run similarly large live trapping campaigns solely dedicated to an assessment of bycatch but there may be the opportunity to include information from further studies (other trap types, habitats, species) in the future.

Most bycatch consisted of taxa not considered endangered. This reflects that endangered species are rare and therefore rarely encountered. There were two species (Eurasian water shrew, Eurasian harvest mouse) trapped that have an early warning status according to the Red List Germany (https://www.rote-liste-zentrum.de/en/Download-Vertebrates-1874.html accessed on 20 December 2021) [66]. The trap success of Eurasian water shrews was the lowest of all species (0.0001 captures per 100 TN) and Eurasian harvest mice occurred in <4% of trapping sessions. Similar to all non-target captures, the seasonal dynamics of the proportion of endangered non-target taxa seemed highest in spring and higher in forests. This indicates that endangered species follow the same capture patterns and seem to utilise traps similar to all non-target species.

There are techniques available to minimise bycatch including bait that is attractive to the target and unattractive to the non-target species. However, many rodent species seem to prefer similar bait or similar bait properties [92,93], which makes it difficult to use a species-specific attractant, but see [36,94]. Trap placement on runways or close to the burrows of target species might limit bycatch but interferes with standardised trapping grids or transect designs. The size of the trap entrance can be minimised to reflect the body size of the target species to exclude all larger taxa. Trap placement away from instead of along margins/habitat interfaces is likely to minimise bycatch but the purpose of the study may require trapping in such locations. Trap sensors are available to indicate capture and they can be used to allow swift removal of trapped animals [44] instead of relying on a fixed trap check frequency. Any method to minimise bycatch needs to be evaluated carefully to make sure that there is no interference with the purpose of the study to guarantee valid result; this is paramount for scientific work. In addition, while maintaining the desired level of animal protection, methods need to be feasible economically regarding workload. This balance might be difficult to find.

## 5. Conclusions

The result presented here can be used to apply protective measures in a targeted fashion and to exclude periods when captures of non-targets and especially endangered species are less probable. Particular attention needs to be paid when trapping in spring along margins if all non-target taxa are to be avoided. For minimising the number and proportion of endangered species, trapping in grassland in spring seems most critical. These times and periods are likely to change when other species are targeted than in this study.

Further parameters potentially related to the risk of trapping non-target species and the risk of mortality of small mammals in traps include temperature, day/night activity and inter-specific competition. Comprehensive data from previous studies should be available to assess such effects and results could be used to further fine-tune measures to minimise the number, proportion and mortality of non-target taxa in live trapping.

## Figures and Tables

**Table 1:** Number of study sites, number of trap nights and number of captures of small mammals in Ugglan live traps from four studies conducted 2010–2021 in six federal states of Germany Baden-Wuerttemberg (BAW), Mecklenburg-Western Pomerania (MWP), North Rhine-Westphalia (NRW), Thuringia (THU), Bavaria (BAV), Lower Saxony (LOS).

Study	Years	Federal State	No. Study Sites				No. Trap Nights				No. Captures			
			Forest	Grassland	Margin	Total	Forest	Grassland	Margin	Total	Forest	Grassland	Margin	Total
# 1			16	8	2	26	39,788	11,172	4067	55,027	9851	1482	519	11,852
	2010–2014	BAW	4	3	0	7	9898	3675	0	13,573	2778	612	0	3390
	2010–2013	MWP	3	1	2	6	8330	2107	4067	14,503	2036	318	519	2873
	2010–2015	NRW	4	3	0	7	9506	3675	0	13,181	2074	181	0	2255
	2010–2013	THU	5	1	0	6	12,054	1715	0	13,769	2963	371	0	3334
# 2	2019–2021	NRW	12	0	0	12	27,500	0	0	27,500	5166	0	0	5166
# 3	2020	BAV	0	0	9	9	0	0	430	430	0	0	937	937
# 4			0	0	12	12	0	0	448.5	448.5	0	0	503	503
	2020	BAV	0	0	4	4	0	0	148.5	148.5	0	0	254	254
	2020	LOS	0	0	8	8	0	0	300	300	0	0	249	249
Total			28	8	23	59	67,538	8109	4945.5	83,355.5	15,017	1482	1959	18,458

**Table 2:** Trap success (captures per 100 TN) and mean (per study site and session) of target and non-target small mammal taxa captured in Ugglan live traps in 28 forest, 8 grassland and 23 margin habitats from four different studies in Germany. Target species in forests and margins were the bank vole (*Clethrionomys glareolus*) and the common vole (*Microtus arvalis*) and in grasslands the common vole (*Microtus arvalis*). No other vertebrates than small mammals were captured, individuals that could not be allocated to a species were not considered further. SE—standard error. * Endangered species according to Red List Germany [66].

Taxon Species		All Habitats		Forest		Grassland		Margin	
		N 100 TN	Mean (±SE)	N 100 TN	Mean (±SE)	N 100 TN	Mean (±SE)	N 100 TN	Mean (±SE)
Cricetidae		16.65	32.38 (±0.14)	17.00	17.21 (±0.05)	11.26	12.11 (±0.40)	24.04	90.46 (±1.09)
Bank vole (*Clethrionomys glareolus*)		13.66	29.46 (±0.15)	15.81	16.59 (±0.06)	0.33	1.68 (±0.28)	14.50	80.50 (±1.24)
Common vole (*Microtus arvalis*)		2.82	23.71 (±0.3)	1.09	13.12 (±0.48)	10.56	12.86 (±0.45)	8.91	35.03 (±0.79)
Field vole (*Microtus agrestis*)		0.15	2.27 (±0.12)	0.09	1.61 (±0.07)	0.3	4.63 (±2.32)	0.63	3.16 (±0.77)
*Microtus* spp.		0.01	0.75 (±0.11)	0.01	0.55 (±0.08)	0.06	0.95 (±0.31)	0	0
Edible dormouse (*Glis glis*)		<0.01	0.41	0.00	0.41	0	0	0	0
Muridae		2.62	21.61 (±0.25)	2.2	4.65 (±0.04)	1.08	4.49 (±0.89)	11.97	59.12 (±1.26)
Yellow-necked mouse (*Apodemus flavicollis*)		1.77	21.46 (±0.31)	1.59	4.24 (±0.05)	0.07	1.09 (±0.39)	8.09	54.81 (±1.35)
Long-tailed field mouse (*Apodemus sylvaticus*)		0.25	11.9 (±0.33)	0.18	1.58 (±0.05)	0.08	1.84 (±1.01)	1.49	23.72 (±0.85)
Striped field mouse (*Apodemus agrarius*)		0.24	3.97 (±0.21)	0.14	2.64 (±0.24)	0	0	2.17	7.3 (±0.86)
*Apodemus* spp.		0.22	2.35 (±0.08)	0.26	2.24 (±0.07)	0.04	0.55 (±0.08)	0.02	11.11
Eurasian harvest mouse * (*Micromys minutus*)		0.14	3.24 (±0.51)	0.01	0.68 (±0.08)	0.90	6.80 (±1.94)	0.18	1.23 (±0.36)
House mouse (*Mus musculus*)		<0.01	1.22	<0.01	1.22	0	0	0	0
House rat (*Rattus rattus*)		<0.01	6.67	0	0	0	0	0.02	6.67
Muste- linae	Least weasel (*Mustela* *nivalis*)	0.02	0.53 (±0.04)	0.02	0.55 (±0.05)	0	0	0.02	0.41
Soricidae		1.19	6.65 (±0.06)	1.03	2.88 (±0.03)	0.92	2.64 (±0.33)	3.98	14.46 (±0.26)
Common shrew (*Sorex araneus*)		0.05	19.14 (±0.93)	0	0	0	0	0.92	19.14 (±0.93)
Eurasian pygmy shrew (*Sorex minutus*)		0.03	10.26 (±0.34)	0	0.41	0	0	0.41	10.92 (±0.32)
*Sorex* spp.		1.08	2.88 (±0.03)	1.03	2.87 (±0.03)	0.92	2.64 (±0.33)	2.10	3.15 (±0.17)
Greater white-toothed shrew (*Crocidura russula*)									
		0.03	17.33 (±0.59)	0	0	0	0	0.49	17.33 (±0.59)
Eurasian water shrew * (*Neomys fodiens*)		0.01	5.05 (±0.81)	< 0.01	0.62 (±0.14)	0	0	0.06	8.00 (±0.00)
Not determined		1.67	6.79 (±0.10)	2.07	6.79 (±0.10)	0	0	0	0
All species		22.14	46.42 (±0.20)	22.32	22.05 (±0.06)	13.27	13.31 (±0.38)	40.02	142.05 (±1.51)

**Table 3:** Overall trap success per season (captures per 100 TN) and mean (per study site and session) of target and non-target small mammal taxa captured in Ugglan live traps in 28 forest, 8 grassland and 23 margin habitats from four different studies in Germany.

Taxon	Season	All Habitats		Forest		Grassland		Margins	
		N 100 TN	Mean (±SE)	N 100 TN	Mean (±SE)	N 100 TN	Mean (±SE)	N 100 TN	Mean (±SE)
Cricetidae	Spring	11.20	36.79 (±0.49)	10.78	10.87 (±0.15)	1.94	2.12 (±0.18)	33.05	97.94 (±2.24)
	Summer	22.25	40.91 (±0.41)	22.94	22.68 (±0.17)	16.20	17.28 (±1.3)	26.49	95.34 (±2.52)
	Autumn	15.93	16.50 (±0.12)	16.8	17.44 (±0.14)	13.13	14.01 (±1.07)	9.84	9.27 (±0.84)
Muridae	Spring	3.88	45.73 (±0.97)	2.37	4.79 (±0.13)	3.64	10.92 (±3.8)	23.44	99.58 (±2.69)
	Summer	1.95	7.77 (±0.14)	2.03	4.38 (±0.11)	0.05	0.41 (±0.00)	4.98	14.61 (±0.5)
	Autumn	2.17	4.71 (±0.10)	2.20	4.78 (±0.13)	0.28	0.98 (±0.17)	7.65	7.23 (±0.93)
Mustelinae	Spring	<0.01	0.41	<0.01	0.41	0	0	0	0
	Summer	0.01	0.41 (±0.00)	0.02	0.41 (±0.00)	0	0	0	0
	Autumn	0.03	0.65 (±0.11)	0.03	0.72 (±0.15)	0	0	0.07	0.41
Soricidae	Spring	0.94	11.51 (±0.28)	0.62	2.31 (±0.11)	0.10	1.22	6.40	18.91 (±0.58)
	Summer	1.42	6.18 (±0.12)	1.26	3.43 (±0.13)	1.81	4.83 (±1.39)	2.62	11.27 (±0.43)
	Autumn	1.19	2.55 (±0.04)	1.19	2.76 (±0.05)	0.67	1.35 (±0.15)	2.92	2.86 (±0.35)
Incognita	Spring	2.02	10.34 (±0.55)	2.46	10.34 (±0.55)	0	0	0	0
	Summer	1.05	4.62 (±0.23)	1.31	4.62 (±0.23)	0	0	0	0
	Autumn	1.98	6.38 (±0.21)	2.46	6.38 (±0.21)	0	0	0	0
All species	Spring	18.03	67.25 (±0.81)	16.23	15.80 (±0.18)	5.68	5.68 (±0.79)	62.90	191.92 (±3.43)
	Summer	26.68	47.34 (±0.44)	27.55	27.32 (±0.19)	18.06	18.06 (±1.26)	34.09	107.61 (±2.58)
	Autumn	21.29	21.16 (±0.14)	22.67	22.59 (±0.18)	14.08	14.17 (±0.99)	20.48	19.43 (±1.39)

## Data Availability

Data supporting reported results can be obtained from the authors. Access to data from studies 3–4 is subject to in-confidence regulation with commercial sponsor.
